# A kinome siRNA screen identifies HGS as a potential target for liver cancers with oncogenic mutations in *CTNNB1*

**DOI:** 10.1186/s12885-015-2037-8

**Published:** 2015-12-29

**Authors:** Frédéric Canal, Elodie Anthony, Aurianne Lescure, Elaine Del Nery, Jacques Camonis, Franck Perez, Bruno Ragazzon, Christine Perret

**Affiliations:** 1 Department Development, Reproduction and Cancer, INSERM U1016, Institut Cochin, 24, rue du Faubourg Saint-Jacques, 75014 Paris, France; 2 CNRS UMR8104, Paris, France; 3 Université Paris Descartes, Sorbonne, Paris Cité, France; 4 Equipe labellisée Ligue Nationale Contre le Cancer, Paris, France; 5 Laboratoire de Génétique et Biologie Cellulaire, Université de Versailles St-Quentin en Yvelines, Montigny le Bretonneux, France; 6 Department of Translational Research, the Biophenics High-Content Screening Laboratory, Institut Curie, PSL Research University, Paris, France

**Keywords:** β-catenin, Synthetic lethality, High throughput screening, Liver cancer, HGS

## Abstract

**Background:**

Aberrant activation of the Wnt/β-catenin pathway is a major and frequent event in liver cancer, but inhibition of oncogenic β-catenin signaling has proven challenging. The identification of genes that are synthetically lethal in β-catenin-activated cancer cells would provide new targets for therapeutic drug design.

**Methods:**

We transfected the parental HuH6 hepatoblastoma cell line with a doxycycline-inducible shRNA against *CTNNB1* (gene coding for β-catenin) to obtain an isogenic cell line pair with or without aberrant β-catenin signaling. Using this hepatoblastoma isogenic cell line pair, we performed a human kinome-wide siRNA screen to identify synthetic lethal interactions with oncogenic *CTNNB1*. The phenotypic readouts of the screen were cell proliferation, cell cycle arrest and apoptosis, which were assessed by image-based analysis. In addition, apoptosis was assessed by flow cytometric experiments and immunoblotting. The potential synthetic lethal relationship between candidates genes identified in the screen and oncogenic *CTNNB1* was also investigated in a different cellular context, a colorectal HCT116 isogenic cell line pair.

**Results:**

We first determined the experimental conditions that led to the efficient expression of shRNA against *CTNNB1* and maximal reduction of β-catenin signaling activity in response to doxycycline treatment. Following high throughput screening in which 687 genes coding for kinases and proteins related to kinases (such as pseudokinases and phosphatases) were targeted, we identified 52 genes required for HuH6 survival. The silencing of five of these genes selectively impaired the viability of HuH6 cells with high β-catenin signaling: *HGS*, *STRADA*, *FES*, *BRAF* and *PKMYT1*. Among these candidates, *HGS* depletion had the strongest inhibitory effect on cell growth and led to apoptosis specifically in HuH6 with high β-catenin activity, while HuH6 with low β-catenin activity were spared. In addition, *HGS* was identified as a potential synthetic lethal partner of oncogenic *CTNNB1* in the HCT116 colorectal isogenic cell line pair.

**Conclusions:**

These results demonstrate the existence of crosstalk between β-catenin signaling and *HGS*. Importantly, HGS depletion specifically affected cells with uncontrolled β-catenin signaling activity in two different types of cancer (Hepatoblastoma HuH6 and colorectal HCT116), and thus may represent a new potential target for novel therapeutic strategies in liver and colorectal cancer.

**Electronic supplementary material:**

The online version of this article (doi:10.1186/s12885-015-2037-8) contains supplementary material, which is available to authorized users.

## Background

Genetic alterations and modifications of the tumor environment often lead to the apparition of weaknesses specific to the tumor that could be exploited therapeutically [1]. For instance, the discovery of synthetic lethal (SL) interactions in cancer cells offers a framework for the design of highly selective drugs. SL interactions occur between two genes when mutation of one gene alone does not alter cellular fitness, but mutation of both causes cell death [2]. Therefore, the discovery of SL interactions involving an “undruggable” oncogene may lead to the identification of new potential therapeutic targets for cancer treatment. This has been well illustrated by the LS interaction between *BRCA* and *PARP1*. Indeed, PARP1 inhibitors show promising activity in clinical trials of breast, ovarian and other cancers associated with *BRCA* mutations [3].

Deregulation of the Wnt/β-catenin pathway, which is a key developmental biology signaling pathway, is a major event in liver cancer and colorectal tumorigenesis [4, 5], which were the 2^nd^ and 4^th^ leading causes of death by cancer worldwide in 2012, respectively (WHO). Indeed, more that 50 % of hepatoblastoma (HB) and a third of hepatocellular carcinoma (HCC) display aberrant activation of Wnt/β-catenin signaling caused by stabilizing mutations of β-catenin in the *CTNNB1* gene [4, 6], while mutations in *APC*, which lead to the ectopic activation of Wnt/β-catenin signaling, are considered the major initiating event in colorectal cancer (CRC) [5, 7]. Thus, the Wnt/β-catenin pathway has become a prime target for cancer research. However, despite intensive research during the past decade, the production of molecules effective against cancers associated with uncontrolled Wnt/β-catenin signaling activity has proven challenging [8]. Hence, the identification of SL partners of oncogenic β-catenin is a promising strategy for the discovery of new therapeutic targets for liver cancer.

Here, we used an siRNA kinome library to perform a high throughput (HT) SL screen of HuH6 isogenic HB cell lines, and we examined the effect of knockdown on cell proliferation, the frequency of mitotic events and induction of apoptosis. The depletion of transcripts of five genes by siRNA led to lethality only in a cellular context characterized by the aberrant activation of the Wnt/β − catenin signaling pathway due to a *CTNNB1* activating mutation. One of these genes (*HGS*) is an SL partner of oncogenic β-catenin in colorectal HCT116 cancer cells, suggesting that the LS interaction between *HGS* and *CTNNB1* is not limited to liver cancer.

## Methods

### Cell culture, transfection and generation of stable shRNA clones

Human hepatoblastoma HuH6 cells were grown in Dulbelcco’s modified Eagle’s medium (DMEM, Gibco, Life Technologies, Carlsbad, CA) with 10 % fetal bovine serum and 100 U/ml penicillin/streptomycin. Colorectal carcinoma HCT116 cells were cultivated in McCoy’s medium, with 10 % fetal bovine serum, at 37 °C in 5 % CO_2_. Parental HuH6 cells were transfected with pTER-β-catenin plasmid using Lipofectamine 2000 (Life Technologies) to generate HuH6^sh*CTNNB1*^ cells [9]. Positive clones were selected following the culture of cells in 5 μg/ml puromycin for 4 weeks. Isolated colonies were picked using cloning rings and clones were amplified for 6 weeks and stored in liquid nitrogen prior to further analysis.

### Reporter assay

The TOPflash/FOPflash reporter plasmids (Millipore, Billerica, MA) were used to determine β-catenin-induced TCF/LEF transcriptional activity. TOPflash is a reporter plasmid containing two sets of three copies of wild-type TCF binding sites driven by the thymidine kinase minimal promoter located upstream from a luciferase reporter gene. FOPflash contains mutated TCF binding sites and is used as a negative control for TOPflash activity. HUH6 and HUH6^sh*CTNNB1*^ were cultivated in the presence or absence of 2 μg/ml of doxycycline for 72 h and transfected with reporter plasmids using Lipofectamine2000 in triplicate in accordance with the manufacturer’s instructions. The pRL-TK plasmid (Promega, Madison, WI) was co-transfected to control for transfection efficiency. Forty-eight hours after transfection, Luciferase activity was measured with the Dual-Luciferase reporter assay system (Promega).

### Real Time quantitative PCR

Total RNA was isolated with TriZol reagent according to the manufacturer’s instructions (Life Technologies). Reverse transcription was performed from 1 μg of total RNA with the Transcriptor First Strand cDNA Synthesis Kit (Roche Diagnostics, Basel, Switzerland) and random hexamer primers. PCR amplification was performed on the LightCycler 480 system with SYBRGreen PCR mix (Roche Diagnostic) and the following primers: HGS forward 5’- CTCCTGTTGGAGACAGATTGGG -3’ and HGS reverse 5’- GTGTGGGTTCTTGTCGTTGAC -3’, 18S forward 5’-GTAACCCGTTGAACCCCATT-3’ and 18S reverse 5’-CCATCCAATCGGTAGTAGCG-3’, CTNNB1 forward 5’- GCTTTCAGTTGAGCTGACCA-3’ and CTNNB1 reverse 5’-GCTTTCAGTTGAGCTGACCA-3’ or Axin2 forward 5’- TGTCTTAAAGGTCTTGAGGGTTGAC-3’ and Axin 2 reverse 5’- CAACAGATCATCCCATCCAACA-3’.

### Transcriptome analysis

After validating RNA quality with the Bioanalyzer 2100 (using Agilent RNA6000 nano chip kit), 50 ng of total RNA was reverse transcribed with the Ovation PicoSL WTA System V2 (NuGEN, San Carlos, CA). Briefly, the resulting double-stranded cDNA was used for amplification based on SPIA technology. After purification according to the manufacturer’s protocol, 2.5 μg of single-stranded DNA was fragmented and labeled with biotin using the Encore Biotin Module (NuGEN). Fragment size was verified with the Bioanalyzer 2100, cDNA was then hybridized to GeneChip® human Gene 1.0 ST (Affymetrix) at 45 °C for 17 h. After overnight hybridization, the chips were washed on the fluidic station FS450 according to the manufacturer’s protocol (Affymetrix, Santa Clara, CA) and scanned with the GCS3000 7G. The image was then analyzed with Expression Console software (Affymetrix) to obtain raw data (cel files) and metrics for quality control. The evaluation of some of these metrics and the distribution of raw data showed no experimental outliers. RMA normalization was performed with R.

### High-content siRNA screening

A kinome siRNA library of 2748 siRNAs targeting 687 genes (four siRNAs per gene) was obtained from Qiagen. We also included a negative siRNA control (anti- luciferase, GL2) and a positive lethal siRNA (anti-KIF11) on each plate. A total of 750 cells were seeded on a 384-well plate (View Plates, Perkin Elmer) in 40 μL of DMEM medium supplemented with 10 % fetal bovine serum and 1 % penicillin/streptomycin. Cells were transfected the day after with 10 nM siRNA using INTERFERin, (0.1 μl per well; Polyplus Transfection, Illkirch, France). Transfection efficiency was estimated by comparing 4,6-diamidino-2-phenylindole (DAPI) staining between cells transfected with negative control siGL2 and those transfected with siKIF11. Cells were fixed 72 h after transfection with 4 % (w/v) formaldehyde for 15 min and washed with phosphate buffered saline (PBS). Cells were next quenched with 0.05 M NH_4_Cl and permeabilized first with a PBS solution containing 0.2 % BSA and 0.05 % saponin, and subsequently with 0.5 % Triton-X100. Cells were then incubated for 60 min with rabbit primary antibody anti-cleaved Caspase 3 (1:500; Sigma-Aldrich) and mouse anti-Ki67 (1:500; Millipore). Next, cells were washed twice in PBS and incubated with Alexa Fluor 488- or Cy3 coupled secondary antibodies (Jackson ImmunoResearch). Nuclei were stained with 0.2 μg/ml DAPI. Images were acquired with an INCell2000 automated wide-field system (GE Healthcare,) at 10x magnification (Nikon 10X/0.45, Plan Apo, CFI/60). Images were analyzed with the INCell Analyzer workstation software (GE Healthcare) and the mean fluorescence intensity of nuclear Ki67 (a proliferation marker) and cleaved-caspase3 antigens was quantified in each cell. Three independent experiments were performed for each treatment.

### Data analysis and hit calling

Positive hits for each gene were identified as follows. Data were first transformed with log or logit functions. B-score normalization was then applied to each replicate, separately, and included corrections for plates, rows and columns [10, 11]. Median and median absolute deviation (MAD) were computed and used to compute Robust Z-scores (RZ-scores) for each sample, according to the formula: score = (value - median)/(1.4826 * Median MAD) [12]. R*Z*-scores were calculated for the comparison of each siRNA against the GL2 negative control population. A gene was identified as a ‘hit’, if the RZ-score for at least two of four siRNAs was > 2 or < -2 in at least two of three replicates. Selected hits were further reordered and validated with four siRNAs per gene (Qiagen), for HUH6 and HUH6^sh*CTNNB1*^ cell lines treated or not treated with 2 μg/ml doxycycline.

### Crystal violet assay

HuH6^sh*CTNNB1*^ cells were cultured with 2 μg/ml of doxycycline for 48 h prior to siRNA transfection and were kept in the presence of doxycycline for the following 72 h. HCTT116, HuH6 and HuH6^sh*CTNNB1*^ cells were plated at 0.1 × 10^6^ cells per well on 6-well plates and transfected with siRNA as indicated. Seventy-two hours after transfection, cells were washed once in PBS and incubated in a crystal violet solution (0.5 % crystal violet, 20 % methanol) for 10 min at room temperature. Plates were washed once with PBS and twice by immersion in water. Cells were then incubated in 1 % SDS solution until the dye was completely solubilized and absorbance was read at 570 nm.

### Apoptosis assay

Cells were harvested and centrifuged at 200 g for 5 min and the pellet was resuspended in 500 μl of culture medium containing 200nM 3,3'-Dihexyloxacarbocyanine iodide (DiOC6_(3)_) and 7.5 μM propidium iodide (PI), (Life Technologies). Cells were incubated for 20 min at 37 °C and samples were analysed using a BD LSR Fortessa flow cytometer (BD Biosciences, San Jose, CA).

### Cell cycle assay

Cells were harvested and fixed with ice cold ethanol 70 % for 20 min, stained with 7.5 μM PI solution containing 100 μg/ml of RNAseI, and then analysis were performed with a BD LSR Fortessa flow cytometer.

### Immunoblot analysis

Cells were washed in ice-cold PBS and lysed in RIPA buffer (Sigma-Aldrich) containing 1X of complete protease inhibitor cocktail (Roche Diagnostics) and centrifuged at 13,000 g for 10 min at 4 °C. Total proteins were resolved by SDS-PAGE, transferred to nitrocellulose and blocked with 5 % BSA. Blots were separately incubated overnight at 4 °C with specific primary antibodies, including 1:1000 anti-HGS/HRS (from Bethyl Laboratories, Montgomery, TX), 1:5000 anti-β-catenin (BD Transduction Laboratories, Franklin Lakes, NJ), 1:5000 anti-γ tubulin and 1:5000 anti-β-Actin (Sigma-Aldrich). After further washing and incubating with corresponding secondary antibodies, the blots were developed by enhanced chemiluminescence (Thermo Fisher Scientific, Waltham, MA).

## Results and discussion

### Identification of liver cancer cell lines with high β-catenin signaling activity

**Fig. 1:**
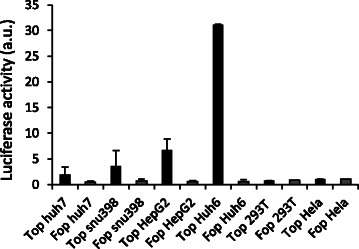
β-Catenin transactivation level in human HCC and HB cell lines. Hepatocellular carcinoma cell lines (HepG2 and SNU398), hepatoblastoma cell lines (HuH6 and HuH7), Human embryonic kidney 293 T cell line and human cervical carcinoma HeLa cell line were transfected with either TOPFlash luciferase (black rows) or FOPFlash luciferase (gray rows) reporter plasmids. Firefly luciferase activity was assessed 24 h after transfection in whole cell extracts and normalized to Renillia luciferase activity

Oncogenic mutations of β-catenin are a frequent event in liver cancer. In this study, we used an siRNA-based loss-of-function screen to identify kinases and proteins related to kinases that are specifically required for the survival of cells in which β-catenin signaling is deregulated. First, we measured β-catenin-induced Tcf/Lef transcriptional activity in liver cancer cell lines with a dual-luciferase assay to identify pertinent cell lines for HT-screening. We selected liver cancer cell lines with alterations in *CTNNB1* (point mutations such as *CTNNB1*^S37C^ and *CTNNB1*^G34V^ found in SNU398 and HuH6 cells, respectively, or a *CTNNB1*^ΔW25_I140^ deletion found in HepG2 cells), and a cell line with a WT *CTNNB1* gene (HCC HuH7 cells). HeLa and Human Embryonic Kidney 293 T cells (HEK293T) were used as negative controls. Only background activity was detected in HeLa and HEK293T cells, whereas HuH7 cells showed modest but significant β-catenin co-transcriptional activity, despite the WT status of β-catenin (Fig. 1), which may be caused by autocrine Wnt signaling in HuH7 cells [13]. Cell lines harboring *CTNNB1* mutations showed great disparity in basal β-catenin signaling activity. SNU398 cells showed rather weak β-catenin transactivation (3.6 times higher than background levels, similar to HuH7 cells), whereas activity was 8- and 36-fold higher than background levels in HepG2 and HuH6 cells, respectively (Fig. 1). Therefore, HuH6 cells were subsequently selected for HT-screening as a reference cell line with high β-catenin signaling activity.

### Generation of a stable HuH6 cell line with doxocycline-inducible sh-RNA against *CTNNB1*

**Fig. 2:**
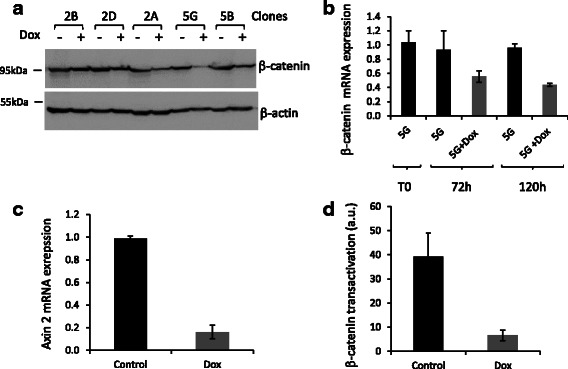
Down regulation of β-catenin expression and inhibition of β-catenin signaling in HuH6^pTER-sh-β-catenin^ transfectant cells. **a** HuH6 cells were transfected with pTER-sh-β-catenin and selected with 2 μg/ml puromycin for 4 weeks. After amplification of isolated colonies, resultant HuH6pTER-sh-β-catenin transfectants were cultivated in the presence or absence of 2 μg/ml doxycycline for 72 h and the abundance of β-catenin in whole cell extracts was assessed by immunoblotting. **b** Clone 5G was cultivated in the presence or absence of 2 μg/ml doxycycline for the indicated times and β-catenin mRNA levels were analyzed by RT-qPCR. **c-** Clone 5G was cultivated in the presence or absence of 2 μg/ml of doxycycline for 72 h and Axin2 mRNA levels were analyzed by RT-qPCR. **d** Clone 5G was cultivated in the presence or absence of 2 μg/ml doxycycline for 72 h and β-catenin/TCF transcriptional activity was assessed by the luciferase reporter assay

We sought to identify SL interactions involving oncogenic β-catenin activity; therefore, we transfected HuH6 cells with pTER-sh-β-catenin encoding a Doxycycline (Dox)-regulated shRNA against *CTNNB1* [9], to obtain HuH6 cells with low β-catenin activity. HuH6 clones containing a stably integrated shRNA vector were selected by culture in the presence of 5 μg/ml puromycin for four weeks. We then analyzed the abundance of β-catenin expression in selected clones treated with Dox by western blotting. The HuH6_5G clone showed the lowest levels of β-catenin (Fig. 2a). The abundance of *CTNNB1* mRNA in the HuH6_5G clone was 40 % lower in cells treated for 5 days with Dox than in control cells, confirming the efficiency of the shRNA (Fig. 2b). Additional experiments showed that β-catenin signaling was strongly impaired in the Dox-treated HuH6_5G clone. RT-qPCR experiments showed that *AXIN2* (a direct target of TCF/LEF/β-catenin complex [14, 15]) mRNA levels were 80 % lower in HuH6_5G cells treated for 5 days with Dox than in untreated (no Dox) control cells (Fig. 2c). In addition, we compared gene expression profiles between untreated and Dox-treated HuH6_5G cells by Affymetrix DNA microarrays: several genes that are known to be activated by β-catenin were significantly down-regulated in cells expressing shRNA against *CTNNB1* (Table 1 and Additional file 1: Table S1), indicating that β-catenin signaling activity was impaired in these cells. Finally, TCF/LEF luciferase reporter activity was substantially lower in Dox-treated HuH6_5G than in control cells (Fig. 2d), confirming that β-catenin signaling is weak when shRNA expression is induced in response to Dox treatment in HuH6_5G cells.

**Table 1:** Target genes activated by Wnt/β-catenin signaling and down-regulated in HuH6^sh*CTNNB1*^ in response to Dox treatment. Gene expression was analyzed with Affymetrix DNA microarrays and the expression profile of HuH6^sh*CTNNB1*^ cells treated with Dox for 2 days was compared with that of untreated HuH6^sh*CTNNB1*^ cells. Note that *CTNNB1* showed a -0.9 Δlog2 in response to shRNA induction with Dox

Wnt target genes	References	ΔLog2
AXIN2	[14, 15]	−1.25
BMP4	[24]	−1.14
NKD1	[25]	−1.13
TNFRSF19	[26]	−1.12
NOTUM	[27]	−1.11
ISM1	[28]	−1.11
LGR5	[29]	−1.03
DUSP6	[30]	−1.02
CCND1	[31]	−0.93
FGF9	[32]	−0.84
MYC	[33]	−0.66
MMP2	[34]	−0.62
HNF1A	[35]	−0.58

Surprisingly, the almost complete inhibition of β-catenin signaling activity did not appear to correlate with β-catenin protein levels (which were only 40 % lower in Dox-treated cells than in control cells). This may be explained by the dual role of β-catenin at the plasma membrane, where it has a structural function in cadherin‐based adherens junctions [16]. Indeed, the remaining β-catenin protein in Dox-treated cells is likely to be trapped in cadherin-based adherent junctions and thus not able to activate genes transcription (β-catenin signaling is close to basal level in Dox-treated cells). Furthermore, we observed that β-catenin signaling was similar between parental HuH6 and untreated-HuH6_5G cells (compare Figs. 1 and 2d). For clarity, the HuH6_5G clone is hereafter named HuH6^sh*CTNNB1*^. Thus, parental Huh6 and HuH6^sh*CTNNB1*^ (without Dox) were used as reference cells with a high β-catenin signaling activity and Dox-treated HuH6^sh*CTNNB1*^ cells were used as reference cells with low β-catenin signaling activity.

The impairment of β-catenin signaling in Dox-treated HuH6^sh*CTNNB1*^ cells was associated with a substantial cell growth defect when cells were seeded at very low density (Additional file 2: Figure S1-A), whereas cell growth was not significantly affected when cells were seeded at medium density (Additional file 2: Figure S1-B). In order to explain the slow increase of Dox-treated HuH6^shCTNNB1^ in culture, we performed cell cycle analysis by measuring DNA content in HuH6^sh*CTNNB1*^ treated or not with Dox by flow cytometry after PI staining (Additional file 2: Figure S1 C-D). We did not detect any significant difference between Dox-treated and untreated HuH6^sh*CTNNB1*^ when seeded at medium density (Additional file 2: Figure S1-D). However, at low cell density we observed an increase in G0/G1 cells in Dox-treated HuH6^shCTNNB1^ when compared to untreated cells, with a concomitant decrease in G2/M cells (Additional file 2: Figure S1-C). These data suggest that G0/G1 phase of cell cycle is longer in Dox-treated HuH6^shCTNNB1^ when seeded at low density. Yet, this modest difference in cell cycle cannot fully explain the drastic decrease in Dox-treated HuH6^shCTNNB1^ growth rate shown in Additional file 2: Figure S1-A. Therefore, we evaluated apoptosis by DiOC6_(3)_ and PI co-staining. DiOC6_(3)_ is a membrane-permeable lipophilic cationic fluorochrome that is used as a probe for mitochondrial transmembrane potential (Δψ_m_). In living cells, DiOC6_(3)_ accumulates in mitochondria and low DiOC6_(3)_ staining reflects a collapse of Δψ_m_. Dissipation of mitochondrial transmembrane potential is a sensitive marker of early apoptotic events. In addition, PI stains only cells whose plasma membrane integrity is compromised. Thus, cells that showed low staining for both DiOC6_(3)_ (loss of Δψ_m_) and PI (loss of plasma membrane integrity) were considered as apoptotic cells. Interestingly, we observed a significant increased in number of apoptotic cells (8.7 % Vs 17.3 %) only in Dox-treated HuH6^shCTNNB1^ when seeded at low density (Additional file 2: Figure S1-E). Thus, prolongated G0/G1 phase and significant increase of apoptosis could explain the slow growth rate of Dox-treated HuH6^shCTNNB1^ when seeded at low density. Interestingly, these results are consistent with those of Chan et al. who showed that the inactivation of mutated *CTNNB1* in the colon cancer HCT116 cell line did not modify the growth of cells passaged under routine conditions, although it significantly decreased colony-forming ability when plated at low density [17].

### HT siRNA screen of HuH6 cells

**Fig. 3:**
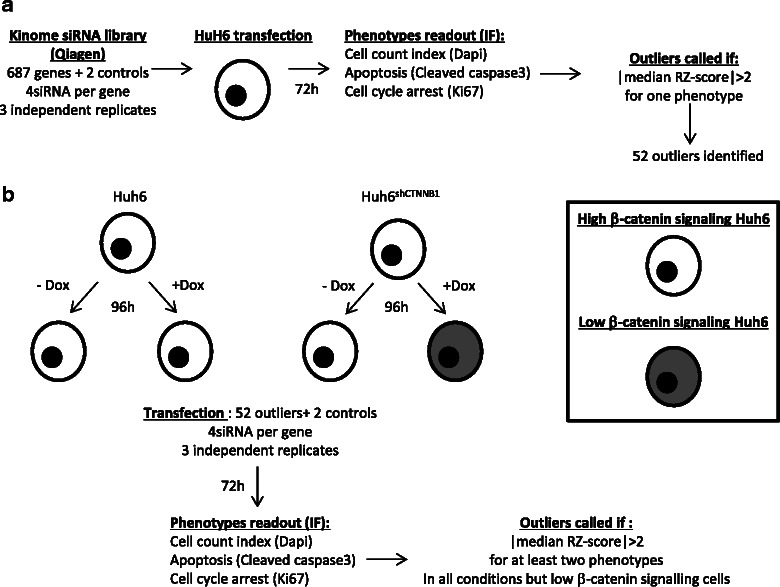
siRNA screen based on immunofluorescence imaging. **a** Identification of kinases required for HuH6 viability. A kinome siRNA library was used to transfect HuH6 cells: 687 genes were individually targeted (four siRNAs per gene) and three independent experiments were performed. Cells were immunolabeled 72 h after transfection with anti-cleaved caspase 3 (apoptosis marker) and anti-Ki67 (cell proliferation markers) antibodies and nuclei were labeled with DAPI (cell count) before the acquisition of images and readout of phenotypes. Three phenotypes were analyzed: (i) cell numbers through DAPI staining, (ii) Apoptosis and (iii) G0-phase arrest with Ki67. All siRNAs with median *Z*-scores >2 or < −2 were considered significant hits, and genes with at least two (out of four) ‘hit’ siRNAs were selected as candidate genes. Following this screen, 52 genes were identified as necessary for HuH6 survival or proliferation. The 52 outliers are listed in Table S2, additional Excel file **b** Counter-screen: Investigation of synthetic lethality relationship between candidate genes and stabilized β-catenin. HuH6 and HuH6^sh*CTNNB1*^ were cultured in the presence or absence of 2 μg/ml doxycycline 72 h, and transfected with a siRNA library including the 52 outliers identified in the primary screen (four siRNAs per gene). Each screen was repeated three times to obtain biological replicates. Transfection was carried out and phenotypes were assessed as described in A. Outliers were called if |robust Z-score| > 2 for at least two phenotypes and two out of four siRNAs, only in cells with high β-catenin signaling. Following the counter-screen, five genes (listed in Table 2) were identified as having a potential lethal synthetic relationship with mutant β-catenin

Kinases play a crucial role in the regulation of Wnt/β-catenin pathway (and in some cases can be targeted by chemicals inhibitors) [18]; therefore, we carried out an siRNA kinome-wide loss-of-function screen (Qiagen, 687 genes - Additional file 3: Table S2) in the HuH6 parental cell line, to identify kinases required for cell viability in cells with high β-catenin (Fig. 3a). Each gene was targeted by four independent siRNAs, resulting in a library of 2748 siRNAs. Positive (KIF11 siRNA) and negative (GL2 siRNA) controls were also added. We examined the effect of silencing on cell growth by high-content immunofluorescence imaging with DAPI staining and specific antibodies against Ki67 (cell proliferation marker) and cleaved caspase 3 (apoptosis).

Each screen was repeated three times to obtain biological replicates. Results are expressed as median *Z*-scores for each siRNA. All siRNAs with a median *Z*-score >2 or < −2 were considered significant hits, and genes with at least two (out of four) ‘hit’ siRNAs were selected as candidate genes (Fig. 3a). Overall, 52 kinases important for cell proliferation and/or cell viability were identified (Additional file 3: Table S2), which represents 7.7 % of identified kinases and associated proteins tested during the screening procedure. These results are consistent with previous reports involving other cancer cell lines [19]. As expected, survival kinases such as those involved in cell cycle progression (e.g. AURKB, CDK3, CDK4, CDK5, CDK7, PLK3, PKMYT1) or in the control of cell growth (e.g. ADK, RPS6KA2) were identified as hits in our primary screen. However, depletion of such cell cycle or cell growth rate related-kinases is likely to be deleterious in wild type cells as well.

### Identification of SL interactions involving oncogenic β-catenin signaling in HuH6 cells

We then carried out an SL counter-screen with siRNA against the 52 survival kinases to identify kinases whose inhibition would affect the viability of HuH6 cells with oncogenic β-catenin activity, but not that of HuH6 cells with low β-catenin activity (sh-induced knock-down of β-catenin). A scheme of the counter-screen is illustrated in Fig. 3b. Briefly, HuH6 and HuH6^sh*CTNNB1*^ were cultivated for 5 days in the presence or absence of Dox, and screened with siRNA targeting the 52 kinases identified in the primary screen. Thus, this counter-screen included three experimental conditions with high β-catenin activity (HuH6, HuH6 + Dox, and HuH6^sh*CTNNB1*^ cells) and one with low β-catenin activity (HuH6^sh*CTNNB1*^ + Dox cells). Proliferation index was slightly lower in HuH6^sh*CTNNB1*^ + Dox cells than in the other three conditions and was strongly influenced by cell density (Additional file 2: Figure S1); therefore, the number of cells/well during the seeding step was optimized to obtain the same number of cells for each condition during the transfection step, with similar growth rate in each experimental condition (Additional file 4: Figure S2). Cell transfection, cell staining, immunofluorescence imaging and statistical analysis were carried out with similar methods used in the primary screen and three independent experiments were performed. Genes were classified as a potential SL partner of the activated form of β-catenin if (i)|median robust Z-score| > 2 for at least two phenotypes in each high β-catenin signaling condition (HuH6, HuH6 + Dox, HuH6^sh*CTNNB1*^), and for at least two siRNAs; and (ii) knockdown had no significant effect (|median robust Z-score| < 2) in cells with low β-catenin signaling (HuH6^sh*CTNNB1*^ + Dox) for all phenotypes analyzed. This counter-screen identified five genes that are potential SL partners of oncogenic β-catenin (Table 2): *HGS* (hepatocyte growth factor-regulated tyrosine kinase substrate), *STRADA* (STE20-related kinase adaptor alpha), *FES* (feline sarcoma oncogene), *BRAF* (v-raf murine sarcoma viral oncogene homolog B) and *PKMYT1* (protein kinase, membrane associated tyrosine/threonine 1).

**Table 2:** List of the five genes identified by HT screening as important for the cellular fitness of high β-catenin signaling cells (HuH6) but not for low β-catenin signaling (HuH6^sh*CTNNB1*^ + Dox) cells. All siRNAs with a median RZ-scores < −2 or >2 were considered hits. Genes were considered as hits if at least two of the four siRNAs were siRNA hits, for at least two phenotypes (cell proliferation, apoptosis or mitotic events) in high β-catenin signaling cells (HuH6, HuH6 + Dox, HuH6^sh*CTNNB1*^), but not in low β-catenin signaling cells (HuH6^sh*CTNNB1*^ + Dox). The RZ-score for the best siRNA hits/gene is shown

	Cell count				Apoptosis				Mitotic events			
Genes Hits	HuH6	HuH6+ Dox	HuH6^shCTNNB1^	HuH6^shCTNNB^ + Dox	HuH6	HuH6+ Dox	HuH6^shCTNNB1^	HuH6^shCTNNB^ + dox	HuH6	HuH6+ Dox	HuH6^shCTNNB1^	HuH6^shCTNNB^ + Dox
HGS	−3.62	−3.11	−3.49		2.33	2.69	2.02				−2.11	
STRADA	−2.64	−4.08	−5.1		3.15	3.11	3.62			3.31		
FES	−2.63	−2.71	−2.44		2.22	2.24	2.11				−2.33	
BRAF	−2.57	−2.83	−2.86		2.77	2.63	2.29					
PKMYT1	−2.51	−2.21	−2.31		3.43	3.98	3.87					

### *HGS* is required for the survival of liver cancer cell lines with oncogenic mutations in *CTNNB1*

**Fig. 4:**
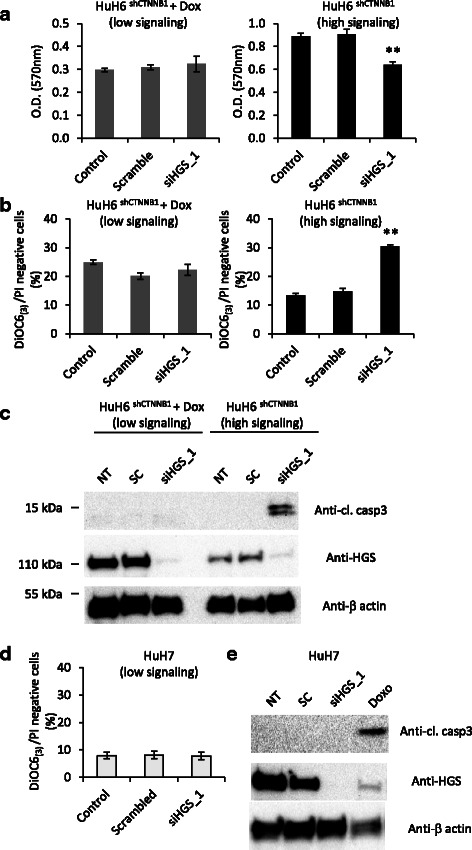
*HGS* knockdown impairs cell viability specifically in hepatoblastoma cells with high β-catenin signaling. **a** HuH6^sh*CTNNB1*^ cells were cultured in the presence or absence of 2 μg/ml doxycycline for 96 h, seeded in 12-well plate and transfected with scramble or siRNA targeting specifically HGS and cultured for 72 h. Untransfected cells (siRNAimax lipofectamine alone) were also included as a control. Cell density was estimated 72 h after cell transfection by crystal violet staining. **b** Cells were cultured and transfected as in A and were stained with DiOC6_(3)_ and PI. Proportion of apoptotic cells was determined by flow cytometric experiment. Note that apoptotic cells showed low labelling for both DiOC6_(3)_ and PI. Means of three independent experiments are shown. c Cells were treated as in A and cleaved caspase3, HGS and β − actin protein levels were examined by immunoblotting. **d** HuH7 were transfected with scramble or siRNA targeting specifically HGS and cultured for 72 h. Untransfected cells (siRNAimax lipofectamine alone) were also included as a control. Proportion of apoptotic cells was determined by flow cytometric experiment after DiOC6_(3)_/IP staining. **e** HuH7 cells were transfected as in D and cleaved caspase3, HGS and β − actin protein levels were examined by immunoblotting. Protein extract from HuH7 treated with 2 μg/ml doxorubicin for 16 h was added as positive control for cleaved caspase3 expression

We investigated biological effect of the five identified candidates. First, we performed the knockdown of *HGS*, *STRADA*, *FES*, *BRAF* and *PKMYT1* by RNAi experiments in HuH6^sh*CTNNB1*^ (high β-catenin signaling) or in Dox-treated HuH6^sh*CTNNB1*^ (low β-catenin signaling) and assessed cell survival by crystal violet staining (Additional file 5: Figure S3). We observed that HGS depletion had the stongest inhibitory effect on cell growth specifically in cells with high β-catenin activity. However, the crystal violet assay is not a sensitive method for the estimation of cell survival. Indeed, we cannot discriminate between adherent living and adherent dead cells with this assay. By flow cytometric experiments, we measured a two-fold increase of apoptotic cells following *HGS* knockdown in high β-catenin signaling HuH6^shCTNNB1^ compared to scramble siRNA, while siRNA against *HGS* did not modify proportion of apoptotic cells in HuH6s^hCTNNB1^ + Dox (Fig. 4b and Additional file 6: Figure S4). These data were confirmed by Western blot analysis of cleaved-caspase 3 expression, a specific marker of apoptotic cells. Indeed, cleaved-caspase 3 was only detected after *HGS* knockdown performed in high β-catenin signaling cells (Fig. 4c). Finally, to show that deleterious effect of *HGS* depletion truly depends on oncogenic β-catenin mutant, we performed *HGS* knockdown in HuH7 which is a hepatoblastoma cell line with wild-type β-catenin. HGS protein expression was efficiently impaired by HGS_1 siRNA in HuH7 (Fig. 4e). However, HGS depletion did not induce apoptosis as indicated by the proportion of DiOC6_(3)_/PI double negative cells measured by flow cytometry (Fig. 4d) and by the absence of cleaved-caspase 3 expression (Fig. 4e). Altogether, our data showed that *HGS* is SL partner of oncogenic β-catenin and its depletion induces apotosis in hepatoblastoma cell lines.

### *HGS* is required for the survival of CRC cell lines with oncogenic mutations in *CTNNB1*

**Fig. 5:**
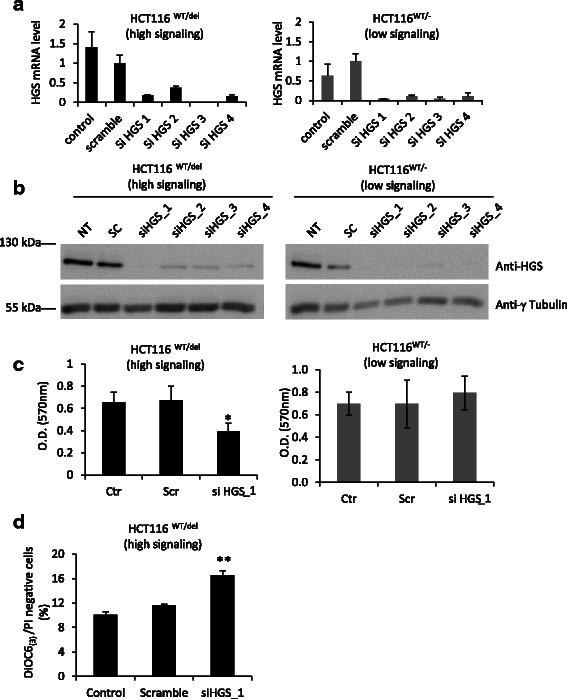
*HGS* knockdown impairs cell viability specifically in HCT116 cells with oncogenic mutations in β-catenin. HCT116^wt/del^ (*CTNNB1*^S45del^ heterozygous) and HCT116^WT/-^ (*CTNNB1*^WT^ only) cells were transfected with scramble or siRNA targeting specifically *HGS* and cultivated for 48 h. Untransfected cells were also included as a control. **a** *HGS* mRNA levels were assessed by RT-qPCR and normalized to the abundance of 18S RNA. The mean ± SD of triplicate samples is presented. **b** The abundance of HGS and γ-tubulin protein was examined by immunobloting. **c** Cell proliferation was visualized by crystal violet staining 72 h after HGS knockdown. Dye was solubilized in 1 % SDS solution and absorbance was read at 570 nm and quantified. The mean ± SD of triplicate samples is presented. d Assessment of apoptosis with DiOC6_(3)_ and IP staining. The mean ± SD of three independent experiments is presented

We decided to analyze SL interactions with oncogenic *CTNNB1* in a different cellular context, the human colorectal cancer cell line HCT116, to determine the specificity of the five potential SL partners of activated-β-catenin. Parental HCT116 is heterozygous for *CTNNB1* (*CTNNB1*^WT/del45^) and has one WT allele and one mutant allele with a 3-bp deletion that eliminates the serine residue at codon 45, leading to synthesis of mutant β-catenin which is constitutively active [17]. In parallel, we used genetically modified HCT116_*CTNNB1*^WT/-^ cells with low β-catenin activity in which the *CTNNB1*^del45^ allele is invalidated [17]. We first performed siRNA experiments for the five targets identified during the SL screen to verify the efficiency of silencing in HCT116 cells by RT-qPCR (Additional file 7: Figures S5). We then estimated cell survival by the crystal violet assay 72 h after transfection of HCT116^WT/del45^ and HCT116^WT/-^ cells with the most efficient siRNA against one of the five potential SL partners of oncogenic β-catenin. Depletion of *STRADA*, *FES*, *BRAF* and *PKMYT1* affected cell survival in both HCT116^WT/del45^ and HCT116^WT/-^, whereas *HGS* knockdown significantly impaired cell survival only in HCT116^WT/del45^ (high β-catenin signaling cells) (Additional file 8: Figure S6). Indeed, *HGS* knockdown efficiently impaired the accumulation of HGS mRNA and protein in both cell lines (Fig. 5a-b); however, the density of HCT116^WT/del45^ cells depleted of *HGS* was 42 % lower than that of control cells, whereas HCT116^WT/-^ cell density was unaffected by *HGS* depletion (Fig. 5c). Moreover, *HGS* knockdown in HCT116^WT/del45^ leads to a significant increase in apoptotic cells number (11.6 % Vs 16.6 %), suggesting that *HGS* expression is required for HCT116^WT/del45^ survival (Fig. 5d). Intriguingly, the abundance of *HGS* mRNA was higher in HCT116^WT/del45^ cells than in HCT116^WT/-^ cells (Fig. 5a), which suggest that *HGS* expression is in part positively regulated by β-catenin in HCT116 cells (directly or indirectly). However, HGS protein levels were not significantly different between HCT116^WT/del45^ and HCT116^WT/-^ cells (Fig. 5b). Thus, the potential regulation of *HGS* by β-catenin cannot fully explain the SL relationship between these two genes. Our results obtained in the colorectal HCT116 cell line support the existence of an SL relationship between *HGS* and oncogenic mutations in β-catenin and identify *HGS* as a potential therapeutic target for cancers with mutations in *CTNNB1*, which is a frequent event in liver cancer.

*HGS* (also called *HRS* for Hepatocyte growth factor-regulated tyrosine kinase substrate) is an essential protein that belongs to multi-vesicular bodies (MVB)/Lysosome complex, which is required to sort membrane proteins and direct them toward either degradation in lysosomes or recycling at the plasma membrane [20, 21]. Interestingly, Toyoshima et al. showed that depletion of *HGS* in fibroblasts led to a defect in E-cadherin degradation and accumulation of E-cadherin at the plasma membrane, which in turn trapped β-catenin at the membrane, preventing its translocation to the nucleus. In addition, *HGS* depletion attenuates the cell growth, tumorigenesis and metastatic potential of Hela cells [22]. Therefore, it is plausible that *HGS* depletion in HuH6 cells leads to the accumulation of E-cadherin at the plasma membrane, which may trap the stabilized form of β-catenin, thus impairing signaling through this pathway and attenuating its oncogenic effect. However, *HGS* is also a key regulator of the molecular machinery involved in receptor sorting and recycling; therefore, *HGS* depletion may affect several signaling pathways. Hence, the exact nature of the SL relationship between oncogenic *CTNNB1* and HGS requires further investigation.

## Conclusions

In conclusion, we performed a siRNA screen that led to the identification of *HGS* as a potential SL partner of oncogenic *CTNNB1*. To ensure high confidence in the hits selected with our approach, we measured three fitness variables (cell proliferation index, induction of apoptosis and number of mitotic events) [23] in two different isogenic cell line pairs from different cellular contexts (liver cancer and CRC). Thus, our findings strongly suggest the existence of a SL interaction between *HGS* and oncogenic *CTNNB1* in HuH6 and HCT116 cells; nonetheless, further investigation is required to validate *HGS* as a new therapeutic target for liver cancers with *CTNNB1* mutations.

### Availability of supporting data

The data sets supporting the results of this article are included within the article and its additional files.

## Additional files

Additional file 1: Table S1. Affymetrix DNA microarray analysis of gene expression in HuH6^sh*CTNNB1*^ cells in response to Dox treatment. Gene expression profile was analyzed with Affymetrix DNA microarrays and the expression profile of HuH6^sh*CTNNB1*^ treated with Dox for 2 days was compared with that of untreated HuH6^sh*CTNNB1*^ cells. (XLSX 1641 kb)
Additional file 2: Figure S1. HuH6^sh*CTNNB1*^ growth rate in response to Dox treatment is dependent of cell density. A-HuH6^sh*CTNNB1*^ cells were cultivated in the presence or absence of 2 μg/ml Dox for 3 days. Cells were then seeded at 3 × 10^3^ cells per well in 12–well plates and cultivated in the presence or absence of 2 μg/ml Dox for 7 days. Cell density was estimated at the indicated times by crystal violet assay and quantified as indicated in Materials and Methods. A representative picture of four independent experiments is shown. Results are expressed as the mean ± S.D. Plain and dashed lines show the growth of HuH6^sh*CTNNB1*^ cells without and with Dox, respectively. B-HuH6^sh*CTNNB1*^ cells were cultivated in the presence or absence of 2 μg/ml Dox for 3 days. Cells were then seeded at 30 × 10^3^ cells per well in a 12–well plate and cultivated in the presence or absence of Dox for 7 days. Cell density was estimated at the indicated times by crystal violet assay. A representative picture of four independent experiments is shown. Results are expressed as the mean ± S.D. Plain and dashed lines show the growth of HuH6^sh*CTNNB1*^ cells without or with Dox, respectively. C- HuH6^shCTNNB1^ cells were cultured in the presence or absence of 2 μg/ml Dox for 3 days and cells were seeded at low density (0.02 × 10^6^ cells/well in 6-well plates) and cultured for another 24 h. Proportion of cells in G0/G1, S or G2/M phases was determined by assessment of DNA content with PI staining and flow cytometry. Black and grey rows represent untreated HuH6^shCTNNB^1 and Dox-treated HuH6^shCTNNB1^ cells, respectively D- HuH6^shCTNNB1^ cells were cultured in the presence or absence of 2 μg/ml Dox for 3 days and cells were seeded at medium density (0. 2 × 10^6^ cells/well in 6-well plates) and cultured for another 24 h. Proportion of cells in G0/G1, S or G2/M phases was determined by assessment of DNA content with PI staining and flow cytometry. Black and grey rows represent untreated HuH6^shCTNNB1^ and Dox-treated HuH6^shCTNNB1^ cells, respectively E- Assessment of apoptosis by flow cytometry with DiOC6_(3)_ and IP staining. Plain black (untreated) and plain grey (2 μg/ml Dox) columns show apoptosis in HuH6^shCTNNB1^ seeded at medium density (0.2 × 10^6^ cells/well), whereas dashed black (untreated) and dashed grey (2 μg/ml Dox) columns show apoptosis in HuH6^shCTNNB1^ seeded at low density (0.02 × 10^6^ cells/well). Means ± S.D. of three independent experiments are presented. (PDF 172 kb)
Additional file 3: Table S2. List of the 52 genes identified among the 687 tested genes in HT screening as important for the cellular fitness of parental HuH6 cells. (XLSX 18 kb)
Additional file 4: Figure S2. HuH6 ± Dox and HuH6^sh*CTNNB1*^ ± Dox growth rates in 384-well plate. Parental HuH6 and HuH6^shCTNNB1^ were cultured in the presence or absence of 2 μg/ml of doxycyclin for 96 h. Parental HuH6 ± Dox and untreated HuH6^shCTNNB1^ were seeded at 750 cells/well while HuH6^shCTNNB1^ + Dox were seeded at 1500 cells/well in 384-well plates. Cell growth rates were assessed at the indicated times after DAPI staining by counting the nuclei in four different fields per well, in 24 wells per condition, acquiered at 10x magnification. Note that HuH6 (plain black line), HuH6 + Dox (dashed black line), HuH6^shCTNNB1^ (plain grey line) and HuH6^shCTNNB1^ + Dox (dashed grey line) showed similar growth rates for at least 72 h. (PDF 89 kb)
Additional file 5: Figure S3. HuH6^shCTNNB1^ cell growth in response to knockdown of potential lethal synthetic hits *HGS*; *STRADA*, *FES*, *BRAF* and *PKMYT1.* HuH6^shCTNNB1^ and Dox-treated HuH6^shCTNNB1^ cells were transfected with scramble siRNA or with siRNAs specifically designed to target *HGS*, *STRADA*, *BRAF*, *PKMYT1or FES*. Untransfected cells were also included as a control. Cell density was estimated 72 h after cell transfection by crystal violet staining, and the effect of siRNA on cell proliferation was determined by the ratio ‘O.D. target siRNA’/ ‘O.D. scramble siRNA’. Gray and black rows show the proliferation rate of Dox-treated HuH6^shCTNNB1^ and HuH6^shCTNNB1^ cells (relative to scamble), respectively. (PDF 91 kb)
Additional file 6: Figure S4. Flow cytometric analysis of apoptotis. Representative DiOC6_(3)_/PI dot plots with typical distribution patterns and gating. Cells gated in P1 were considered as apoptotic cells. Cells with high PI labelling were considered as necrotic cells (loss of plasma membrane integrity). Cells with low PI labelling and high DiOC6_(3)_ labelling were considered as living cells. (PDF 252 kb)
Additional file 7: Figure S5. Validation of siRNA efficiency in HCT116 cells. HCT116^WT/S45del^ cells were transfected with scramble siRNAs or with four different siRNAs designed to target *HGS*, *STRADA*, *FES*, *BRAF* or *PKMYT1*. Untransfected cells were also included as a control. Forty-eight hours after transfection, the mRNA levels of these genes were assessed by RTqPCR and normalized to the abundance of 18S RNA. (PDF 196 kb)
Additional file 8: Figure S6. HCT116^WT/S45del^ and HCT116^WT/-^ cell growth in response to knockdown of potential lethal synthetic hits *HGS*; *STRADA*, *FES*, *BRAF* and *PKMYT1*. HCT116^WT/S45del^ and HCT116^WT/-^ cells were transfected with scramble siRNA or with siRNAs specifically designed to target *HGS*, *STRADA*, *FES*, *BRAF* or *PKMYT1*. Untransfected cells were also included as a control. Cell density was estimated 72 h after cell transfection by crystal violet staining, and the effect of siRNA on cell proliferation was determined by the ratio ‘O.D. target siRNA’/ ‘O.D. scramble siRNA’. Gray and black rows show the proliferation rate of HCT116^WT/S45del^ and HCT116^WT/-^ cells (relative to scamble), respectively. Note that cell proliferation was affected by the silencing of each genes, both in HCT116^WT/S45del^ and HCT116^WT/-^ cells, except for *HGS*, the depletion of which affected specifically HCT116^WT/S45del^ cells. (PDF 92 kb)

## Abbreviations

SL: Synthetic lethal
HCC: Hepatocellular carcinoma
HB: Hepatoblastoma
CRC: Colorectal cancer
siRNA: Small interfering RNA
shRNA: short hairpin RNA
HT: High throughput
Dox: Doxycyclin
MVB: Multivesicular bodies
TCF/Lef: T-cell factor/Lymphocyte enhancer factor.
