# Supplementary figures and images for: A kinome siRNA screen identifies HGS as a potential target for liver cancers with oncogenic mutations in CTNNB1
Source: BMC Cancer. 2015 Dec 29;15:1020. doi: 10.1186/s12885-015-2037-8 (PMC4696130; doi:10.1186/s12885-015-2037-8)

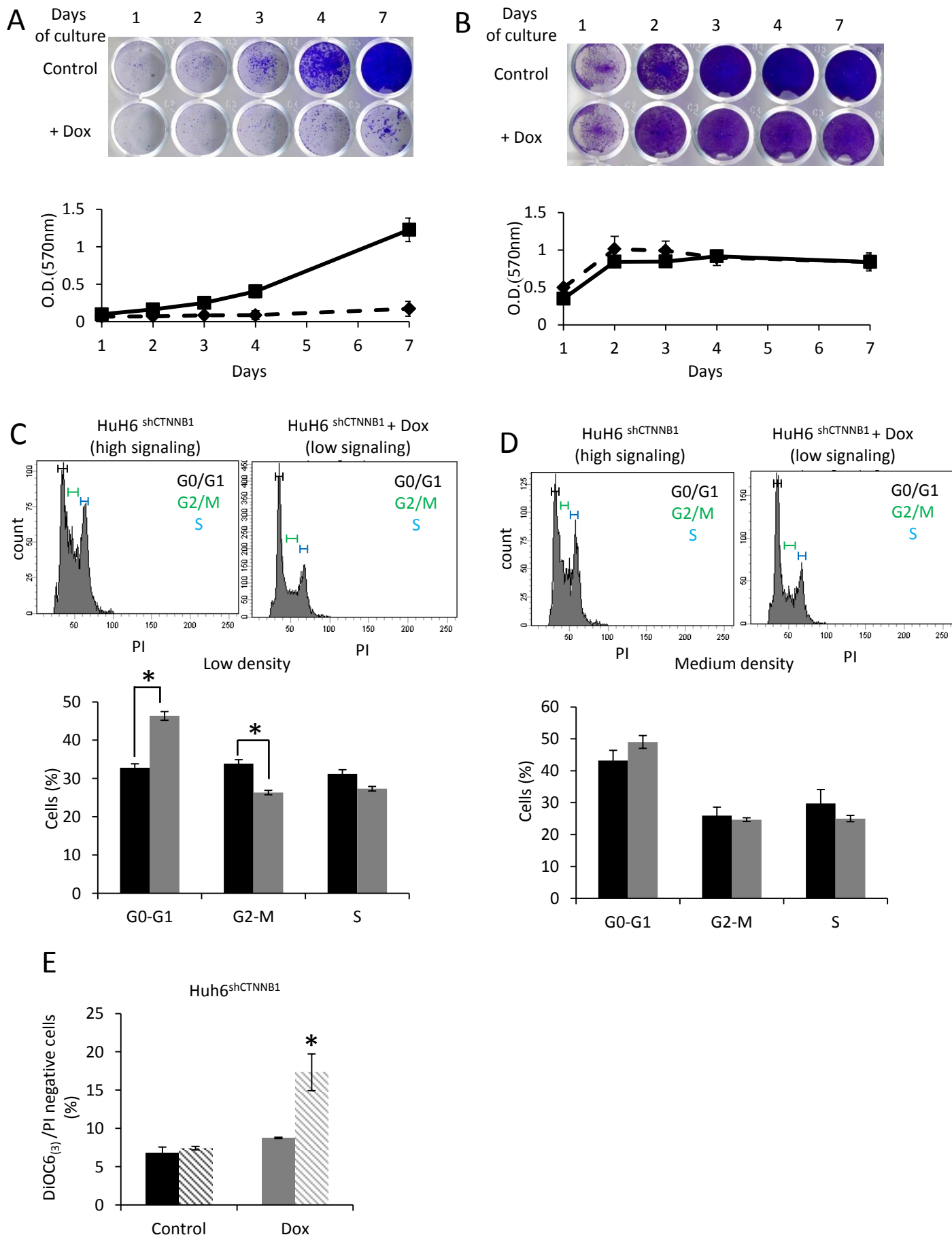

Figure S1

Supplement: Additional file 1: Table S1. — Affymetrix DNA microarray analysis of gene expression in HuH6shCTNNB1 cells in response to Dox treatment. Gene expression profile was analyzed with Affymetrix DNA microarrays and the expression profile of HuH6shCTNNB1 treated with Dox for 2 days was compared with that of untreated HuH6shCTNNB1 cells. (XLSX 1641 kb) [file 12885_2015_2037_MOESM1_ESM.xlsx]

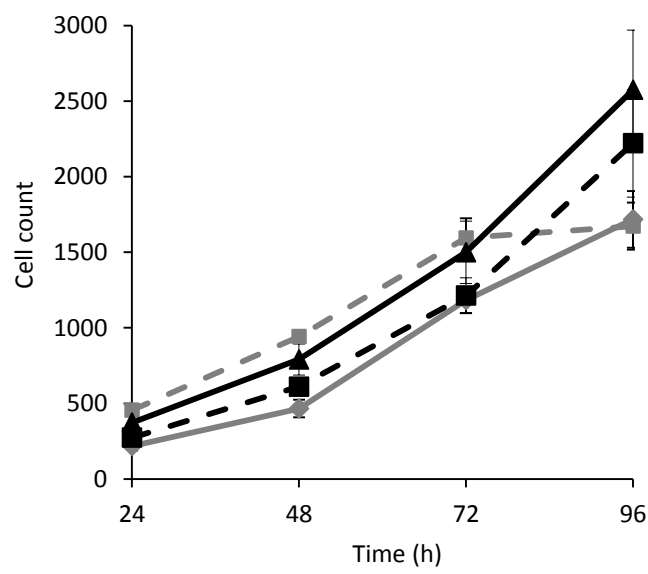

Figure S2

Supplement: Additional file 2: Figure S1. — HuH6shCTNNB1 growth rate in response to Dox treatment is dependent of cell density. A-HuH6shCTNNB1 cells were cultivated in the presence or absence of 2 μg/ml Dox for 3 days. Cells were then seeded at 3 × 10^3^ cells per well in 12–well plates and cultivated in the presence or absence of 2 μg/ml Dox for 7 days. Cell density was estimated at the indicated times by crystal violet assay and quantified as indicated in Materials and Methods. A representative picture of four independent experiments is shown. Results are expressed as the mean ± S.D. Plain and dashed lines show the growth of HuH6shCTNNB1 cells without and with Dox, respectively. B-HuH6shCTNNB1 cells were cultivated in the presence or absence of 2 μg/ml Dox for 3 days. Cells were then seeded at 30 × 103 cells per well in a 12–well plate and cultivated in the presence or absence of Dox for 7 days. Cell density was estimated at the indicated times by crystal violet assay. A representative picture of four independent experiments is shown. Results are expressed as the mean ± S.D. Plain and dashed lines show the growth of HuH6shCTNNB1 cells without or with Dox, respectively. C- HuH6shCTNNB1 cells were cultured in the presence or absence of 2 μg/ml Dox for 3 days and cells were seeded at low density (0.02 × 10^6^ cells/well in 6-well plates) and cultured for another 24 h. Proportion of cells in G0/G1, S or G2/M phases was determined by assessment of DNA content with PI staining and flow cytometry. Black and grey rows represent untreated HuH6shCTNNB1 and Dox-treated HuH6shCTNNB1 cells, respectively D- HuH6shCTNNB1 cells were cultured in the presence or absence of 2 μg/ml Dox for 3 days and cells were seeded at medium density (0. 2 × 10^6^ cells/well in 6-well plates) and cultured for another 24 h. Proportion of cells in G0/G1, S or G2/M phases was determined by assessment of DNA content with PI staining and flow cytometry. Black and grey rows represent untreated HuH6shCTNNB1 and Dox-treated HuH6shCTNNB1 cells, respective [file 12885_2015_2037_MOESM2_ESM.pdf]

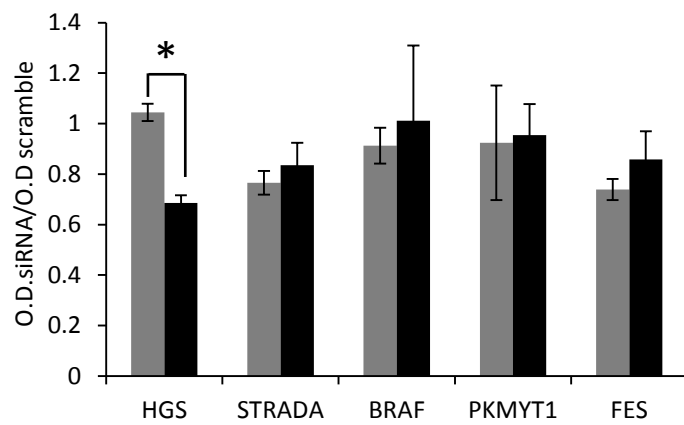

Figure S3

Supplement: Additional file 3: Table S2. — List of the 52 genes identified among the 687 tested genes in HT screening as important for the cellular fitness of parental HuH6 cells. (XLSX 18 kb) [file 12885_2015_2037_MOESM3_ESM.xlsx]

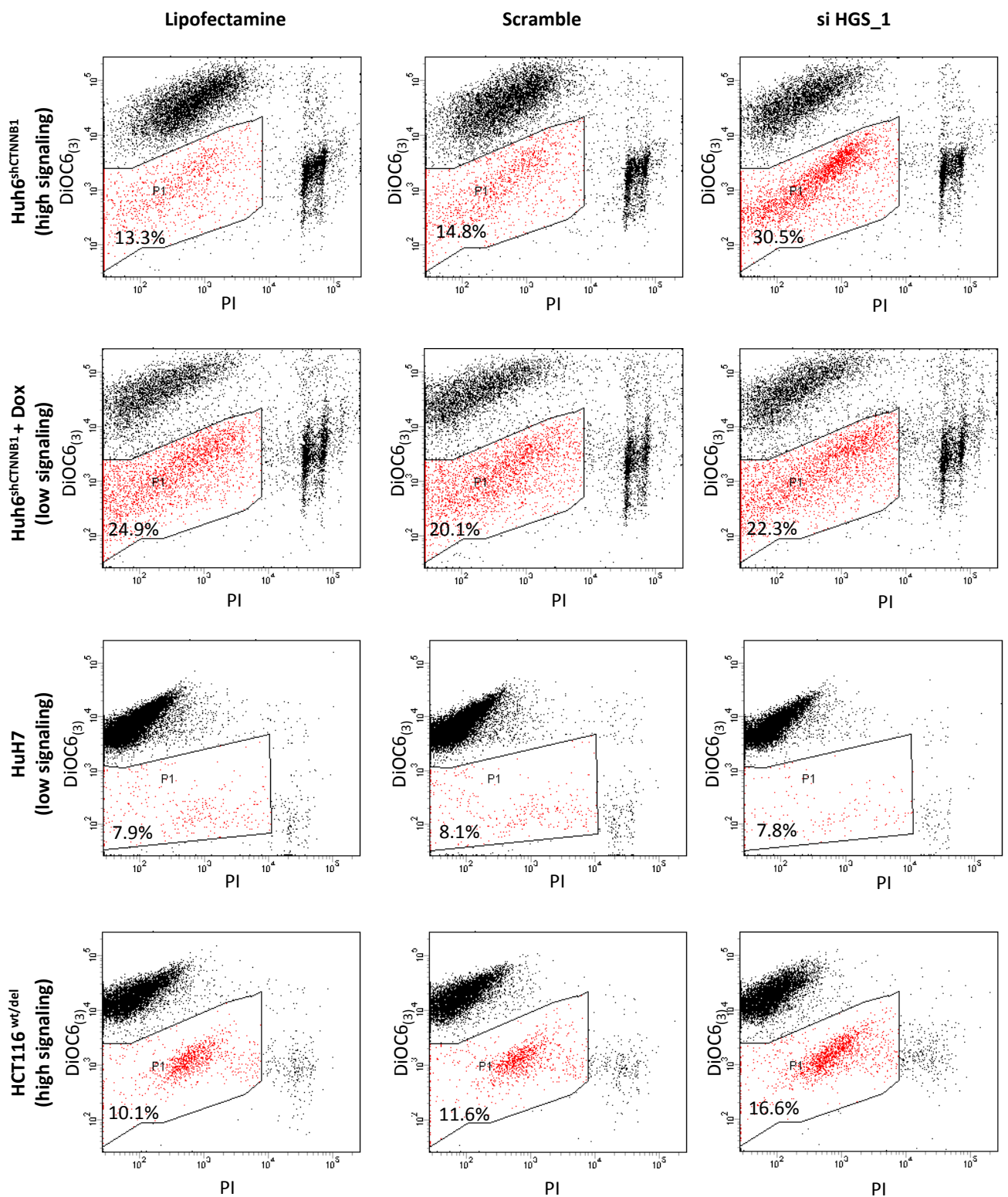

Figure S4

Supplement: Additional file 4: Figure S2. — HuH6 ± Dox and HuH6shCTNNB1 ± Dox growth rates in 384-well plate. Parental HuH6 and HuH6shCTNNB1 were cultured in the presence or absence of 2 μg/ml of doxycyclin for 96 h. Parental HuH6 ± Dox and untreated HuH6shCTNNB1 were seeded at 750 cells/well while HuH6shCTNNB1 + Dox were seeded at 1500 cells/well in 384-well plates. Cell growth rates were assessed at the indicated times after DAPI staining by counting the nuclei in four different fields per well, in 24 wells per condition, acquiered at 10x magnification. Note that HuH6 (plain black line), HuH6 + Dox (dashed black line), HuH6shCTNNB1 (plain grey line) and HuH6shCTNNB1 + Dox (dashed grey line) showed similar growth rates for at least 72 h. (PDF 89 kb) [file 12885_2015_2037_MOESM4_ESM.pdf]

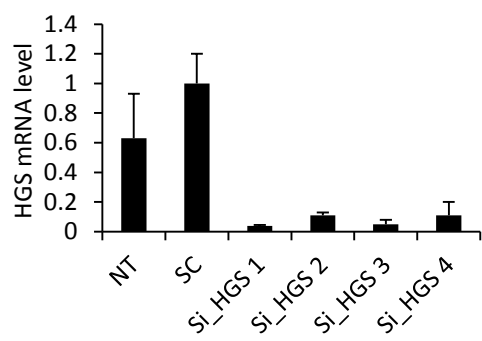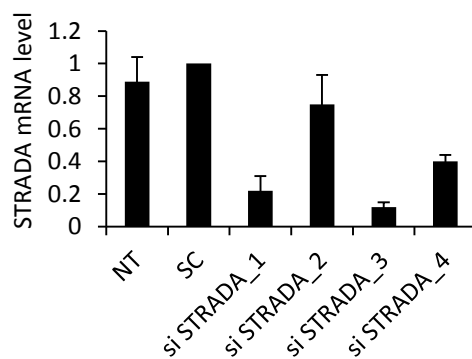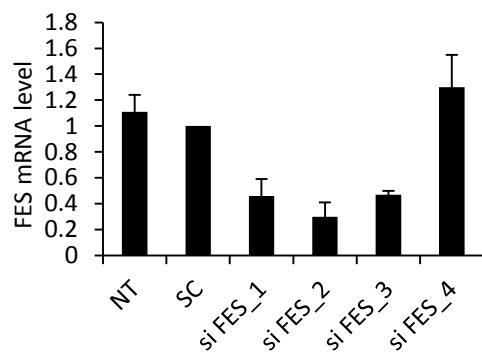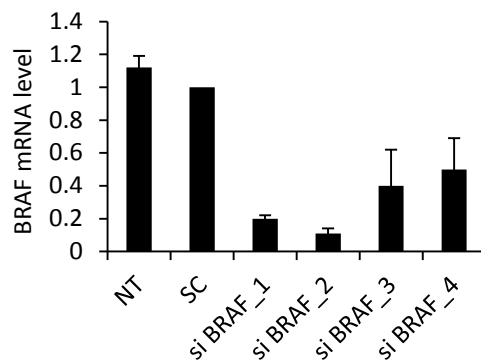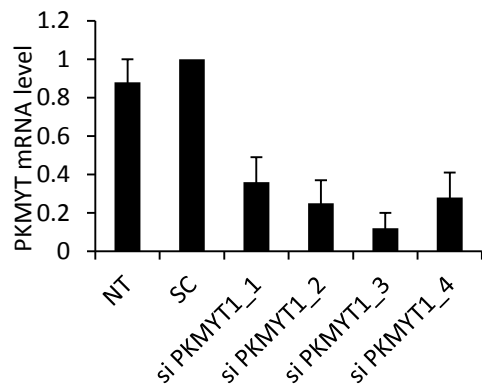

Figure S5

Supplement: Additional file 5: Figure S3. — HuH6shCTNNB1 cell growth in response to knockdown of potential lethal synthetic hits HGS; STRADA, FES, BRAF and PKMYT1. HuH6shCTNNB1 and Dox-treated HuH6shCTNNB1 cells were transfected with scramble siRNA or with siRNAs specifically designed to target HGS, STRADA, BRAF, PKMYT1or FES. Untransfected cells were also included as a control. Cell density was estimated 72 h after cell transfection by crystal violet staining, and the effect of siRNA on cell proliferation was determined by the ratio ‘O.D. target siRNA’/ ‘O.D. scramble siRNA’. Gray and black rows show the proliferation rate of Dox-treated HuH6shCTNNB1 and HuH6shCTNNB1 cells (relative to scamble), respectively. (PDF 91 kb) [file 12885_2015_2037_MOESM5_ESM.pdf]

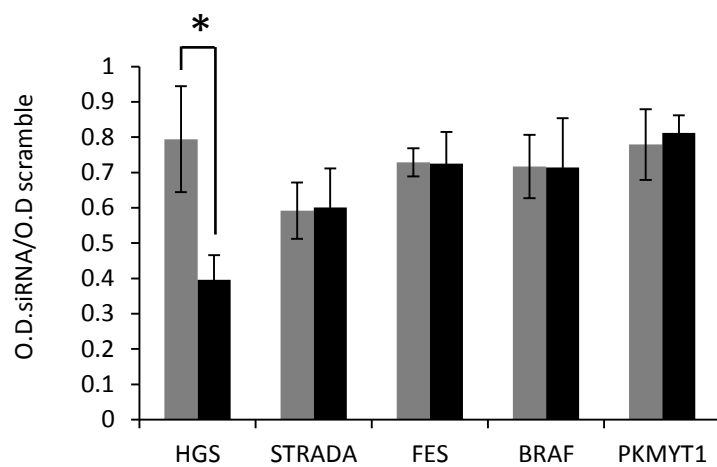

Figure S6

Supplement: Additional file 6: Figure S4. — Flow cytometric analysis of apoptotis. Representative DiOC6(3)/PI dot plots with typical distribution patterns and gating. Cells gated in P1 were considered as apoptotic cells. Cells with high PI labelling were considered as necrotic cells (loss of plasma membrane integrity). Cells with low PI labelling and high DiOC6(3) labelling were considered as living cells. (PDF 252 kb) [file 12885_2015_2037_MOESM6_ESM.pdf]
